# Ticks and Associated Pathogens From Rescued Wild Animals in Rainforest Fragments of Northeastern Brazil

**DOI:** 10.3389/fvets.2020.00177

**Published:** 2020-04-08

**Authors:** Maísa Santos Fonseca, Thiago Campanharo Bahiense, Aretha Alves Borges Silva, Valeria Castilho Onofrio, Thiago Doria Barral, Barbara Maria Paraná Souza, Rejane Maria Lira-da-Silva, Ilka Biondi, Roberto Meyer, Ricardo Wagner Portela

**Affiliations:** ^1^Laboratório de Imunologia e Biologia Molecular, Instituto de Ciências da Saúde, Universidade Federal da Bahia, Salvador, Brazil; ^2^Laboratório de Parasitologia Veterinária, Instituto de Ciências da Saúde, Universidade Federal da Bahia, Salvador, Brazil; ^3^Laboratório Especial de Coleções Zoológicas, Instituto Butantan, São Paulo, Brazil; ^4^Mestrado em Medicina e Bem-Estar Animal, Universidade Santo Amaro, São Paulo, Brazil; ^5^Escola de Medicina Veterinária e Zootecnia, Universidade Federal da Bahia, Salvador, Brazil; ^6^Núcleo Regional de Ofiologia e Animais Peçonhentos da Bahia, Departamento de Zoologia, Instituto de Biologia, Universidade Federal da Bahia, Salvador, Brazil; ^7^Laboratório de Animais Peçonhentos e Herpetologia, Departamento de Ciências Biológicas, Universidade Estadual de Feira de Santana, Feira de Santana, Brazil

**Keywords:** *Amblyomma* spp., Anaplasmataceae family, *Hepatozoon* spp., *Ixodes loricatus*, *Rickettsia* spp., wildlife animals

## Abstract

The Ixodidae family comprises ticks that are hematophagous ectoparasites and are considered vectors of several hemoparasites from the Anaplasmataceae family and the genus *Hepatozoon, Babesia*, and *Rickettsia*. These ectoparasites parasitize domestic and wild animals belonging to several vertebrate groups. Ticks are highly adapted to different biomes and thus possess a wide geographical distribution. In the Brazilian state of Bahia, localized in the Northeast region, there are large rainforest fragments. Studies have rarely been carried out on ticks, and their hemoparasites, that parasitize wild animals in this region. Thus, this study aimed to identify the tick species parasitizing wild animals rescued in rainforest fragments of Bahia and investigate the presence of hemoparasites in tick tissues. During a 2-year period, 238 ticks were collected from 41 wild mammalians, reptiles, and amphibians. These ectoparasites were taxonomically classified according to their morphological characteristics. The ticks identified belonged to five different species from the Ixodidae family: *Amblyomma varium, Amblyomma rotundatum, Amblyomma nodosum, Ixodes loricatus*, and *Rhipicephalus sanguineus*. For the first time, an *A. rotundatum* parasitizing the *Mesoclemmys tuberculata* turtle was described. PCR assays using DNA extracted from salivary glands or midgut of the ticks were performed to detect specific DNA fragments of hemoparasites from the genus *Rickettsia, Ehrlichia, Babesia, Hepatozoon*, and from the Anaplasmataceae family. The results showed positive detection of the *Rickettsia* genus (7.9%), Anaplasmataceae family (15.8%), and *Hepatozoon* genus (15.8%). Specific DNA from the *Ehrlichia* and *Babesia* genera were not detected in these samples. Specific DNA from members of the Anaplasmataceae family was detected in *A. varium* for the first time. The present work showed that amphibians, reptiles, and mammals from Bahia's Atlantic Forest areparasitized by different tick species, and that these ectoparasites present pathogens in their tissues that impact both humans and animals due to their zoonotic potential.

## Introduction

Ticks are hematophagous ectoparasites belonging to the phylum Arthropoda with significant medical and veterinary importance due to their role in the transmission of pathogens to humans, domestic, and wild animals (1). The tick-borne pathogens disseminated to vertebrate hosts include protozoa belonging to the *Hepatozoon* and *Babesia* genera and bacteria belonging to the Anaplasmataceae family and the *Rickettsia* genus (2, 3).

The number and diversity of tick-borne disease cases have been increasing due to anthropization, growth in both tick population, and in the number of potential hosts, as well as important changes in the environment, and improvement of diagnostic methodologies (4). Bacteriosis and protozoonosis carried by these vectors are related to productivity losses in livestock, financial losses, and damage to the health of human populations near periurban forest fragments (5). As human activities, directly and indirectly, affect wildlife habitats and niches, some diseases that were formerly restricted to wildlife are being spread and becoming emergent diseases in domestic animals and humans (6).

In the neotropical region, the ixodids are represented by ~117 species divided into five genera: *Amblyomma, Ixodes, Rhipicephalus, Dermacentor*, and *Haemaphysalis* (7–9). Recent records of the Ixodidae family in Brazilian tick fauna recorded 48 different species, 33 of which were from the *Amblyomma* genus (9, 10). *Amblyomma* spp. were found parasitizing a wide diversity of hosts that include humans, other mammalians, avians, reptiles, and amphibians (11). In Brazil, nine species carried ticks from the *Ixodes* genus, and two species carried ticks from the *Rhipicephalus* genus (9).

The study of the infection by protozoa and bacteria that are transmitted by ticks can provide clinical support for each host species, such as the knowledge of the symptoms related to each agent. Also, the study of the ixodofauna in a particular region is vital for public health due to the large number of etiological agents, vectors, and hosts involved in the epidemiology of tick-borne diseases (5). It is crucial to investigate diseases that affect wild animals for the preservation of these species (12). Likewise, the potential risk to human and animal health underlines the importance of studies that focus on tick-associated pathogens (13).

Thus, the aim of this study was to identify ixodids obtained from wild animals from rainforest fragments of the Atlantic Forest in Northeastern Brazil and to detect in these ticks the presence of *Rickettsia, Ehrlichia*, and Anaplasmataceae bacteria; and *Babesia* spp. and *Hepatozoon* spp. protozoans.

## Materials and Methods

### Study Area and Sample Collection

All tick collections were performed during a 2-year period in three different localities: (a) in the Center for Wildlife Rescue and Triage (CETAS-IBAMA), (b) in the Center of Ophiology and Venom Animals of the Federal University of Bahia (NOAP/UFBA), both located in the municipality of Salvador, and (c) in the Laboratory of Venom Animals and Herpetology of the State University of Feira de Santana (LAPH/UEFS), located in the municipality of Feira de Santana. These three locations receive animals that were rescued by the Environmental Police or civilians in periurban rainforest fragments. Right after the rescue process and in the moment that the animals were entering the conservation centers, the animals were examined for tick infestations, and ectoparasites were collected. The ticks collected from wild specimens were stored in 70% ethanol until identification and DNA extraction. The Chico Mendes Institute of Biodiversity (ICMBio), from the Brazilian Ministry of Environmental Issues approved this study (SISBIO 52141-2). The animals were screened, identified, and put into quarantine in accordance with Brazilian law.

### Tick Identification

Tick identification was performed as previously described by Onofrio et al. (7) for adults and Martins et al. (14) for nymphs. Identifications were performed in double-blind assays at the Laboratory of Veterinary Parasitology (ICS/UFBA) and the Laboratory of Zoological Collections of the Butantan Institute. A stereoscope coupled to a digital camera was used to register the main structures used for the tick identifications. After taxonomic identification, the ticks were hermetically sealed into flasks containing 70% ethanol.

### Tick Dissection

For DNA extraction from isolated organs, specifically, salivary glands and midgut, adult male and female ticks of sufficient size were dissected as described by Edward et al. (15) in a modified protocol. Briefly, the ticks were fixed with entomological pins in paraffin-filled Petri dishes. After fixing, the ticks were covered with a sodium phosphate buffer solution (pH 7.4). Under a stereomicroscope, the dissection procedure started with a lateral incision of the body, followed by separation of the ventral and dorsal parts, exposing the internal organs. The salivary glands were withdrawn before the midgut to avoid contamination. Subsequently, both organs were washed in phosphate buffer saline (PBS) pH 9.6 and preserved in 70% ethanol until molecular evaluation.

### DNA Extraction and Molecular Analysis of Pathogens

PCR assays using genus-specific primers were performed to investigate the presence of pathogens of the *Rickettsia, Ehrlichia, Babesia*, and *Hepatozoon* genera and the Anaplasmataceae family. DNA samples were obtained from tick salivary glands or midguts, or the whole tick if the tick were too small for dissection.

DNA extraction was performed using the PureLink™ Genomic DNA Mini kit (Invitrogen®) as described by the manufacturer. When a host animal had more than one tick, a pool of the samples (salivary gland, midgut, or whole tick) was used for DNA extraction. The PCR assays used genus-specific primers and PCR experimental conditions previously described: for identification of the *Rickettsia* genus, primers CS78-F (5′-GCAAGTATCGGTGAGGATGTAAT-3′) and CS323-R (5′-GCTTCCTTAAAATTCAATAAATCAGGAT-3′) were used to amplify a gltA gene fragment of 401 bp (16); for the *Ehrlichia* genus, primers DSB720-F (5′-ATTTTTAGRGATTTTCCAATACTTGG-3′) and DSB720-R (5′-CTATTTTACTTCTTAAAGTTGATAWATC-3′) were used to amplify a dsb gene fragment of 349 bp (17); for the Anaplasmataceae family, the forward primer was GE2 (5′-GTTAGTGGCAGACGGGTGAGT-3′) and the reverse primer was HE3 (5′-TATAGGTACCGTCATTATCTTCCCTAT-3′) that amplify a ribosomal 16S gene fragment of 360 bp (18); for the *Babesia* genus, primers BAB143-167-F (5′-CCGTGCTAATTGTAGGGCTAATACA-5′) and BAB694-667-R (5′-GCTTGAAACACTCTARTTTTCTCAAAG-3′) were used to amplify a ribosomal 18S gene fragment of 551 bp (19); finally, for the *Hepatozoon* genus, primers HEP142-169-F (5′-GGTAATTCTAGAGCTAATACATGAGC-3′) and HEP743-718-R (5′-ACAATAAAGTAAAAAACAYTTCAAAG-3′) were used to amplify a ribosomal 18S gene fragment of 574 bp (17, 19). A negative control using ultra-pure water and a positive control. Genomic DNA purified from the blood of dogs infected with *Hepatozoon canis, Ehrlichia canis, Babesia canis, Anaplasma platys*, as confirmed by nucleotide sequencing, and from a *Rickettsia parkeri* reference strain, were used as positive controls. PCR products were marked with SYBR™ Green Master Mix (ThermoFisher Scientific^©^) and submitted to agarose gel (1.5%) electrophoresis to verify the presence or absence of the specific amplicons and to confirm or dismiss the presence of the pathogens in tick sample DNA.

## Results

### Morphological Identification of Ticks and Their Hosts

In this study, 238 ticks were collected from 41 wild reptiles, amphibians, and mammalians rescued in rainforest fragments from Northeastern Brazil (Table 1). Regarding the life stage of all ticks identified, 85.7% were adults (204/238), represented by 87.7% (179/204) females and 12.3% (25/204) males, 13.0% (31/238) were nymphs and only 1.3% (3/238) were larvae (Table 2). Although for *Amblyomma nodosum* and *Amblyomma varium* both males and females were collected, there were no *Amblyomma rotundatum* males (Table 2). Regarding *Ixodes loricatus* only adults, male and female, were collected (Table 2).

**Table 1:** Host class, species, and number as it relates to tick species and number per animal(s) subdivided by host capture location.

**Class**	**Host**	***n***	**Tick**	***n***	**Animal Capture Location**
Mammalian	*Bradypus torquatus*	1	*Amblyomma varium*	5	Mata de São João
		1		2	Camaçari
		2		4	Salvador
	*Tamandua tetradactyla*	3	*Amblyomma nodosum*	21	Salvador
	*Cavia aperea*	1	*Rhipicephalus sanguineus*	1	Salvador
	*Didelphis aurita*	3	*Ixodes loricatus*	3	Mata de São João
		2		4	Salvador
	*Didelphis albiventris*	3		23	Salvador
			*Ixodes* sp.	2	
Reptile	*Boa constrictor*	5	*Amblyomma rotundatum*	43	Salvador
		1		1	Candeias
		2		27	Feira de Santana
		1		1	Mata de São João
	*Eunectes murinus*	2		2	Salvador
		1		8	Lauro de Freitas
		1		1	Feira de Santana
	*Python* spp.	1		4	Lauro de Freitas
	*Helicops carinicaudus*	1		72	Salvador
	*Waglerophis merremii*	1		1	Salvador
	*Micrurus lemniscatus*	1		1	Salvador
	*Bothrops leucurus*	4		6	Salvador
	*Iguana iguana*	1		1	Mata de São João
	*Mesoclemmys tuberculata*	1		2	Camaçari
Amphibian	*Rhinella jimi*	2	*Amblyomma rotundatum*	2	Ipecaetá
			*Amblyomma* sp.	1	
	TOTAL	41	TOTAL	238	

**Table 2:** Larvae, nymph, and adult ticks found in different animals from Northeastern Brazil rainforest fragments.

**Hosts**	**Ticks**						
	***A. rotundatum***	***A. nodosum***	***A. varium***	***I. loricatus***	***R. sanguineus***	***Amblyomma sp*.**	***Ixodes* sp**.
**Mammalian**							
*B. torquatus*			7M 4F				
*C. aperea*					1F		
*D. albiventris*				2M 7F 14N			2L
*D. aurita*				2M 5F			
*T. tetradactyla*		14M 7F					
**Reptile**							
*I. iguana*	1F						
*B. constrictor*	56F 16N						
*B. leucurus*	6F						
*E. murinus*	11F						
*H. carinicaudus*	72F						
*M. lemniscatus*	1N						
*Python* sp.	4F						
*W. merremii*	1F						
*M. tuberculata*	2F						
**Amphibian**							
*R. jimi*	2F					1L	

*F, female adult ticks; M, male adult ticks; N, nymphs; L, larvae*.

The morphological identification of adults and nymphs (235/238) showed the presence of five species of ticks: *A. rotundatum, A. nodosum, A. varium, I. loricatus*, and *Rhipicephalus sanguineus* (Table 1). Three larvae (3/238) were found, and due to the absence of an identification key to classify larval species, the larvae were only classified by genus. One larva, found on *Rhinella jimi*, was identified as *Amblyomma* sp. and two larvae found on *Didelphis albiventris* as *Ixodes* sp. (Table 1). The morphological characteristics observed in each tick that allowed species identification are presented in Supplementary Figures 1–5.

The *Amblyomma* genus was the most abundant, representing 86.1% (205/238) of all ticks collected. *A. rotundatum* was the most prevalent tick species with a frequency of 72.3% (172/238) (Table 1). All ticks of this species were collected from either reptiles or the amphibian *R. jimi*. All reptiles rescued in this study were infested exclusively by *A. rotundatum* (Table 1).

Among snakes in our study, *B. constrictor* and *Helicops carinicaudus* had the highest infestation rate. The ticks obtained from these species contributed 83.7% of all *A. rotundatum* collected (144/172), with 41.9% (72/172) coming from each species of snake (Table 1). However, on *B. constrictor*, ticks were collected from 10 animals, while on *H. carinicaudus* all ticks were collected from one animal with a high degree of parasitism (Table 1). Additionally, *A. rotundatum* infested five additional snake species in this study: *Eunectes murinus* (11/172), *Bothrops leucurus*, (6/172), *Python* spp. (4/172), *Waglerophis merremii* (1/172), and *Micrurus lemniscatus* (1/172) (Table 1). Two more reptiles were infested by *A. rotundatum*, one *Iguana iguana*, and one *Mesoclemmys tuberculata* tortoise (Table 1).

The other tick species identified belonging to the *Amblyomma* genus were *A. nodosum* and *A. varium* with frequencies of 8.8% (21/238) and 4.6% (11/238), respectively (Table 1). Adults of *A. nodosum* and *A. varium* were found parasitizing mammals of the species *Tamandua tetradactyla* and *Bradypus torquatus*, respectively (Tables 1, 2).

Adults and nymphs of *I. loricatus* were found only in the mammalian hosts *Didelphis aurita* and *Didelphis albiventris*, accounting for 12.6% (30/238) of ticks collected. These two species of marsupials represent 50% of all infested mammals examined in this study (Table 1). The one specimen of *R. sanguineus* found in this study (0.4%; 1/238) was collected from *Cavia aperea* (Table 1).

### Identification of Pathogens in Tick DNA Samples

A total of 36 DNA samples from *A. rotundatum, A. nodosum, A. varium*, and *I. loricatus* were obtained and evaluated for the presence of pathogens (Table 3). Our results showed no amplification for *Babesia* spp. or *Ehrlichia* spp. DNA in all samples tested. *Rickettsia* spp. were detected in three samples obtained from midgut or whole ticks. Of these three positives amplicons for *Rickettsia* spp., two originated from *A. nodosum* collected on *T. tetradactyla*, and one from *A. varium* collected on *B. torquatus* (Table 3). *A. rotundatum* and *I. loricatus* collected from reptile and amphibian hosts presented negative results for detection of *Rickettsia* spp. DNA.

**Table 3:** Relationship between hosts, ticks, tick organs, and pathogens.

**Host class**	**Host species**	**Tick species**	**Organ**	**Location**	**Pathogen**
Mammalians	*B. torquatus*	*A. varium*	Whole tick	Camaçari	*Rickettsia*
			Whole tick	Salvador	None
			Salivary gland	Camaçari	None
			Gut	Camaçari	None
			Salivary gland	Mata de São João	None
			Gut	Mata de São João	Anaplasmataceae
			Whole tick	Mata de São João	None
			Whole tick	Salvador	None
			Whole tick	Feira de Santana	None
	*T. tetradactyla*	*A. nodosum*	Gut	Salvador	*Rickettsia*
			Whole tick		*Rickettsia*
			Whole tick		None
			Whole tick		None
			Salivary gland		None
			Gut		None
			Salivary gland		None
			Gut		None
	*D. albiventris*	*I. loricatus*	Salivary gland	Salvador	None
			Gut		None
			Whole tick		None
			Whole tick		None
Reptiles	*B. constrictor*	*A. rotundatum*	Salivary gland	Salvador	*Hepatozoon*
			Gut		*Hepatozoon*
			Salivary gland	Feira de Santana	None
			Gut		Anaplasmataceae
			Salivary gland	Salvador	*Hepatozoon*
			Gut		*Hepatozoon*
	*M. tuberculata*	*A. rotundatum*	Salivary gland	Camaçari	None
			Gut		Anaplasmataceae
	*E. murinus*	*A. rotundatum*	Gut	Feira de Santana	None
	*Python spp*.	*A. rotundatum*	Salivary gland	Lauro de Freitas	None
			Gut		None
	*H. carinicaudus*	*A. rotundatum*	Salivary gland	Salvador	None
			Gut		Anaplasmataceae/*Hepatozoon*
Amphibians	*R. jimi*	*A. rotundatum*	Salivary gland	Ipecaetá	Anaplasmataceae/*Hepatozoon*
			Gut		Anaplasmataceae

DNA from the Anaplasmataceae family was identified in six (6/36) samples originating from ticks collected on mammalian, reptile, and amphibian hosts (Table 3). One of the positive samples originated from the midgut of *A. varium* collected from *B. torquatus* (1/36), four originated from midgut of *A. rotundatum* collected from various species [*M. tuberculata* (1/36), *B. constrictor* (1/36), *H. carinicaudus* (1/36), and *R. jimi* (1/36)], and one from the salivary glands of *A. rotundatum* collected on *R. jimi* (1/36). *Hepatozoon* spp. DNA was positively amplified in six of the 36 tick samples. Positive amplicons originated from both midgut and salivary glands DNA samples from *A. rotundatum* that had infested two *B. constrictor* snakes (4/36), from the midgut of one specimen collected on *H. carinicaudus* (1/36), and from salivary glands of another specimen collected on *R. jimi* (1/36) (Table 3). Co-infection of *Hepatozoon* spp. and the Anaplasmataceae family were found in midgut DNA samples from *A. rotundatum* collected on *H. carinicaudus* and from salivary glands of *A. rotundatum* infesting *R. jimi*.

DNA samples obtained from different organs, salivary glands and midgut, differed in amplification for pathogens in relationship to the species of tick. Pathogens were only identified in salivary glands from *A. rotundatum*. For all other ticks species, DNA samples only showed amplification of pathogen DNA in samples from the midgut or whole tick (Table 3).

## Discussion

### Hosts and Ticks From Northeast Brazilian Rainforest Fragment

Several studies have described ticks from the *Amblyomma* genus parasitizing reptiles, amphibians, avians, and mammalians (7, 20, 21). To date, 23 tick species have been identified in the Amazon region, with *Amblyomma* being the most prevalent genus (22). The state of Mato Grosso, located in the Midwestern region of Brazil, composed by the Amazon, Cerrado, and Pantanal biomes, showed a tick diversity of 27 species with *Amblyomma* once again being the most prevalent (23). Our results also found *Amblyomma* ticks to be the most prevalent, especially in reptile and amphibian hosts.

In the state of Pernambuco in the Northeast region of Brazil, *A. rotundatum* seems to be the most prevalent tick species infesting reptiles (24). Various reptile species and toads belonging to the *Rhinella* genus in the Amazon were infested by *A. rotundatum* (25). In the state of Mato Grosso, three species of *Rhinella, R. bergi, R. marina*, and *R. schneideri* were infested with adults and nymphs of *A. rotundatum* and *Amblyomma dissimile*, with *A. dissimile* being the most frequent tick species found (23). The amphibian *R. jimi* rescued in this study were parasitized with *A. rotundatum* and one larvae of the *Amblyomma* genus. It has been previously described that *Rhinella* are predominately infested by ticks from *A. rotundatum* and *A. dissimile* species (26). In Brazil, the infestation by *A. rotundatum* on *R. jimi* was previously described in the states of Pernambuco (13, 27) and Ceará (28).

Snakes are frequently found parasitized by ticks (13, 29–31). The snakes of our study were all infested with *A. rotundatum* ticks. A previous evaluation of tick parasitism in snakes from a fragment of the Atlantic Forest showed *A. rotundatum* was the most prevalent, representing 99.1% of all ticks collected (29). However, other tick species, most frequently *A. dissimile*, are found infesting different snake species (23, 25, 31). Species from the Boidae family represented 50.1% of reptile species rescued in Salvador, Bahia, between 2012 and 2014 with *B. constrictor* making up 40.2% of these rescued reptile species. Rescue of wildlife animals in urban and periurban areas could be associated with the expansion of cities and increased deforestation, as these actions reduce biodiversity and require surviving native species to adapt to other environments (32). This high incidence of *B. constrictor* in periurban environments may explain the high prevalence of this snake species in our study. Pontes et al. (29) evaluated nine specimens of *Helicops carinicaudus* and found no tick infestations. However, the one *H. carinicaudus* captured in our study had the highest infestation rate among all animals rescued, showing a different pattern of parasitism between *H. carinicaudus* and hard ticks than previously reported.

With the exception of *B. constrictor* and *H. carinicaudus*, in our study, *A. rotundatum* infested snakes at lower rates than previously reported. In a study conducted in Venezuela, six specimens of *E. murinus* were found infested with *A. dissimile*, but not *A. rotundatum* (33). In the Northern (34), Midwest (23), and Southern region (35) of Brazil, specimens of *E. murinus* were found infested by *A. dissimile*. The parasite-host relationship between *B. leucurus* and *A. rotundatum* has already been described in the state of Bahia (36). *A. rotundatum* was also found infesting *B. leucurus* and *Bothrops erythromelas* in the state of Ceará (28). The only record in Brazil of *A. rotundatum* infestation in the *Python* genus occurred in a *Python molurus bivittatus* (30). The authors characterized this *Python* species as an artificial host of *A. rotundatum* due to the exotic origin of this snake. Snakes from the *Python* genus are exotic species on the American continent (37). However, *A. rotundatum* has also been described to infest *P. bivittatus* in the state of Florida in the United States, where both the tick and snake are exotic species (38). In experimental conditions, *A. rotundatum* ticks were able to establish an artificial infestation in *W. merremii* (39), but, in natural environments, this interaction was not observed. Until now, *Micrurus lemniscatus* has not been described as a host snake species for *A. rotundatum*. Thus, this study describes a novel parasite-host relationship, although another species from this genus, *Micrurus ibiboboca*, has been shown to be parasitized by *A. rotundatum* in the Northeast region of Brazil (28, 30).

Although the majority of the reptiles examined in this study were snakes, the lizard, *I. iguana*, and tortoise, *M. tuberculata*, were also infested with *A. rotundatum*. Infestation in *I. iguana* by this species of tick has already been registered in the states of Pernambuco and Paraíba (24, 30). In contrast, in the Midwestern region of Brazil, 44 *I. iguana* specimens from either free-living or captivity niches were only infested by *A. dissimile* (23). Although *A. rotundatum* has already been described to infest *Mesoclemmys vanderhaegei* in the Midwestern region of Brazil (23), this is the first record of parasitism of this species of tick on *M. tuberculata*.

Adult forms of *A. nodosum* have been described in mammalian hosts, almost exclusively on anteaters, as on *T. tetradactyla* and *M. tridactyla*, while immature forms are frequently found on birds hosts (23, 40, 41). Infestation by *A. nodosum* in avians was previously described for different species (23, 42, 43). *A. nodosum* occurrence was described in Brazilian's Southeastern (44), Midwestern (40), Northern (25), and Northeastern region (30, 43). An infestation by *A. nodosum* was found on *T. tetradactyla* in the state of Ceará (45) and on *M. tridactyla* and *T. tetradactyla* in the state of Pernambuco (30).

Adult *A. varium* display high host specificity, found almost exclusively in sloths from the Bradypodidae family, such as *Bradypus tridactylus, Bradypus variegatus, B. torquatus* (46). *A. varium* infestation has also been described in the *Choloepus hoffmanni* and *Choloepus didactylus* sloth species (25, 46). Interestingly, in Colombia, *A. varium* was described to parasitize a domestic dog (47). The occurrence of *A. varium* in Brazil has been registered in the Southeastern, Northeastern, Midwestern, and Northern regions (22, 23, 43, 46, 48). In the Northeastern region, specimens of *A. varium* were found infesting *B. torquatus* in the state of Bahia (46) and *B. variegatus* in the state of Paraíba (30).

Our study found *I. loricatus* on *D. aurita* and *D. albiventris* as previously described in diverse Brazilian states such as Paraná, Rio Grande do Norte, São Paulo, and Pernambuco (21, 49–51). In the state of Pernambuco, *I. loricatus* was found infesting five marsupial species, including *D. aurita* and *D. albiventris*, with high infestation frequencies in these two marsupial species (50), similar to that observed in our study.

*Rhipicephalus sanguineus* mainly parasitizes domestic dogs and is considered an important vector for pathogens that have negative impacts on human and veterinary health (1). The majority of studies involving *R. sanguineus* have been in domestic animals, but this tick species is also found on wild animals (30, 52, 53). In Brazil, adults and nymphs of *R. sanguineus* have previously been found to parasitize black-tailed marmosets [*Mico melanurus*; (23)], and the carnivorous species *Nasua nasua, Cerdocyon thous, Chrysocyon brachyurus, Pseudalopex vetulus*, and *Leopardus tigrinus* (23, 30, 52, 54). However, the parasitism of *R. sanguineus* in *C. aperea* is described for the first time in this study.

*Amblyomma rotundatum* is described as a parthenogenetic tick species (55, 56) and the majority of the studies (13, 24, 30, 57) have only encountered female's ticks, as was observed in our study. However, four *A. rotundatum* males were reported in Brazil, in the Amazonian state of Rondônia (44, 58) and in the USA (59). Adults, both female and male, and nymph stages of *A. varium* (46) and *A. nodosum* (41) have been described to infest different mammalians. However, the life cycle and biology of these species is not well known in nature.

It is noteworthy to say that the stress level caused by the rescue, or even the particular situations that led the animals to be rescued, are important factors to be considered for the presence or not of these ectoparasites. This is a complex issue, and further studies made through an active collect of these animals in their own habitat would be valuable to verify *in situ* such host-parasite relationships.

### Tick-Borne Associated Pathogens

The detection of pathogens in the tissues of ticks can provide relevant information about parasite-hosts relationships and their associated pathogens. In performing analysis of different organs (midgut and salivary glands), we aimed to provide information about the location of the parasite in the vector body. The midgut of ticks are the main organ involved in the acquisition of blood-borne pathogens, and the salivary glands have an important role in the transmission of parasites during blood uptake mediated by the tick bite (60). Diverse pathogens are described as being transmitted by saliva; for example, protobacteria belonging to the Anaplasmataceae family are mainly transmitted by hard ticks via saliva as ticks feed (61). Experiments have shown that tick saliva is capable of enhancing infection in vertebrate hosts for several pathogens (60), including *Rickettsia conorii* (62).

The *Babesia* genus comprises apicomplexa parasites that are naturally transmitted by ixodids (63). However, the literature associates *Babesia* transmission with ticks from at least four genera: *Rhipicephalus, Ixodes, Haemaphysalis*, and *Hyalomma* (64–66). In Brazil, ticks belonging to the *Amblyomma* and *Ixodes* genera collected from small mammals were negative for *Babesia* pathogens (67). Negative PCR amplification for *Babesia* DNA was observed in samples extracted from *Amblyomma cajennense* and *Amblyomma ovale* collected from dogs in Pantanal, Brazil (68). However, *Babesia caballi* was found infecting *Amblyomma variegatum* ticks isolated from cattle in the Republic of Guinea (69). Recently, one female tick of *Amblyomma testudinarium* collected from a dog in Taiwan was positive for *Babesia gibsoni*, but *R. sanguineus* and *Heamaphysalis hystricis* are the species most frequently infected by *Babesia* (70). Thus, ticks belonging to the *Amblyomma* genus may not be the main vectors of tick-borne pathogens from the *Babesia* genus.

Our samples showed positive PCR amplification for *Rickettsia* spp. only in DNA samples extracted from ticks collected from mammalian hosts. *Rickettsia rickettsii* and *Rickettsia parkeri* are causative species of tick-borne spotted fever in Brazil, and these pathogens were found in this country in ticks collected from capybaras in Mato Grosso state (71) and from dogs in Bahia state (72). *Rickettsia* bacteria have been reported in ticks infesting several wild mammalian species throughout Brazil (73–76), and in Brazilian wild birds (77, 78). *Rickettsia parkeri and R. bellii* have been found in *A. nodosum* collected from *M. tridactyla* and *T. tetradactyla* in the Southeast and Central-West regions of Brazil (76). One *A. rotundatum* collected in the Amazonian region of Brazil showed positive PCR results for *R. belli* (79). Although our PCR results showed no amplification of *Rickettsia* spp. DNA in samples collected from ticks infesting amphibians, a previous study of *A. rotundatum* collected from *R. jimi* from the Northeastern region in Brazil, detected Rickettsia in 100 % of tick DNA samples (27). For reptilian hosts, we again detected no PCR amplification for *Rickettsia* spp. DNA, but this hemopathogen has already was described in ticks collected from a tortoise, *Chelonoidis denticulata*, in the state of Espírito Santo in Southeastern Brazil (48). *Rickettsia bellii* was also found in *A. rotundatum* and *A. dissimile* ticks collected from snakes in several regions of Brazil (80).

For the first time, this study detected DNA from members of the Anaplasmataceae family in *A. varium*. Members of the *Anaplasma* and *Ehrlichia* genera are the causative agent of anaplasmosis or ehrlichiosis diseases in both domestic and wild animals and in humans (81). The Anaplasmataceae family was detected by PCR of *Ornithodoros spheniscus* ticks collected in Chile (82). However, the sequencing approach was unable to distinguish between *Candidatus Neoehrlichia* or organisms of the *Anaplasma* and *Ehrlichia* genera (82). In Northern Brazil, amplicons for the *Anaplasma* genus were detected in two *A. dissimile* collected from *Bothrops atrox* snakes (80). The genus *Ehrlichia*, belonging to the Anaplasmataceae family, are the most investigated pathogen of this family. However, in our results, although there was positive PCR amplification for the Anaplasmataceae family, there was no positive PCR amplification specifically for the *Ehrlichia* genus. The most studied species of this genus are *Ehrlichia canis* and *Ehrlichia chaffeensis* (83). In studies that, focused on wildlife, *Ehrlichia* spp. were found affecting terrestrial mammalian carnivores in a number of countries worldwide (84). *Ehrlichia ruminantium* was detected in *Amblyomma* ticks collected from tortoises in Kenya (85) and in the United States from animals imported from Zambia (86). In addition, DNA from *E. canis* was found in *Amblyomma latum* collected from monitor lizards in Kenya (85).

Six midgut and salivary glands from *A. rotundatum* samples garnered positive PCR result for *Hepatozoon* in our study. *Hepatozoon* spp. are the most common blood parasite found in reptiles (87). These hemoparasites affect reptiles on almost all continents of the world. However, description of *Hepatozoon* in ticks collected from reptiles are rare. Ticks belonging to the *Amblyomma* genus collected from snakes in Thailand, presented with a high prevalence of *Hepatozoon* infection (96%) (88). *Amblyomma* sp. ticks collected from a python in Australia were positive for *Hepatozoon* PCR amplicons (89). *Hepatozoon* sp. have only recently been found in Brazilian reptiles, specifically in two *A. dissimile* ticks collected from *Bothrops atrox* snakes in the Northern region of Brazil (80). In our work, we showed the presence of *Hepatozoon* sp. in ticks from two different snakes, *B. constrictor* and *H. carinicaudus*. The role of these ticks in transmission of *Hepatozoon* genus in snakes (80), and other reptiles was not determined. Experimental ingestion of rodent tissues infected with *Hepatozoon ayorgbor* by snakes successfully transmitted this parasite (90), suggesting that the feed of infected animals can be an important pathway in horizontal transmission of *Hepatozoon* sp.

Finally, we observed PCR amplification for *Hepatozoon* spp. and Anaplasmataceae family DNA in salivary glands in *A. rotundatum*, characterizing the first description of the co-infection of these hemopathogens in this tick species. The co-infection of different hemopathogens are expected once a host is infected by multiple pathogens (91). Descriptions of multiple tick-borne pathogens in the same tick specimen are increasing. Recently, in one *Ornithodoros atacamensis* tick from Chile, PCR detected three different pathogens from the Anaplasmataceae family and the *Borrelia* and *Hepatozoon* genera (82). In several *Ixodes ricinus* ticks, a predominant European tick species, co-infection of *Anaplasma phagocytophilum* and *Rickettsia helvetica* were found in both the salivary glands and midgut of males and females (92). In *I. ricinus* a co-infection with *Borrelia burgdorferi*, a causative agent of Lyme disease, and *Babesia microtti* has been described (93). In an investigation of pathogens in *Amblyomma americanum*, a tick species common in the United States, a co-infection of *Rickettsia amblyommii* and *Ehrlichia chaffeensis* in one tick and of *R. amblyommii* and *Ehrlichia ewingii* in two additional ticks was detected (94). In Brazil, an *R. sanguineus* tick collected from a dog was positive for *E. canis* and *Leishmania infantum* flagellate protozoa PCR amplicons (95). Our description of a co-infection with *Hepatozoon* spp. and Anaplasmataceae family pathogens in the midgut and salivary glands of *A. rotundatum* ticks collected from *H. carinicaudus* and *R. jimi* represents the first description of this double pathogen infection in the Brazilian territory.

In conclusion, our findings described known and new information about tick species and their respective hosts in Atlantic Forest fragments in Northeastern Brazil. The presence of several pathogen species in ectoparasite tissues provides new information that is important in human and veterinary medicine due to their zoonotic potential.

## Data Availability Statement

All datasets generated for this study are included in the article/Supplementary Material.

## Ethics Statement

The animal study was reviewed and approved by The Chico Mendes Institute of Biodiversity (ICMBio), from the Brazilian Ministry of Environmental Issues (SISBIO 52141-2).

## Author Contributions

TCB, VO, RM, and RP designed the study. AS and TDB conducted the fieldwork. MF, AS, BS, and TDB conducted the laboratory work. IB and RL-d-S received and maintained the rescued snakes from which ticks were collected. MF, TCB, and RP wrote the manuscript. VO, IB, BS, RM, and RL-d-S revised the manuscript. All authors approved the final manuscript.

### Conflict of Interest

The authors declare that the research was conducted in the absence of any commercial or financial relationships that could be construed as a potential conflict of interest.
