# Supplementary material for: Ticks and Associated Pathogens From Rescued Wild Animals in Rainforest Fragments of Northeastern Brazil
Source: Front Vet Sci. 2020 Apr 8;7:177. doi: 10.3389/fvets.2020.00177 (PMC7179698; doi:10.3389/fvets.2020.00177)
Supplement: Supplementary file 1 [file Data_Sheet_1.PDF]

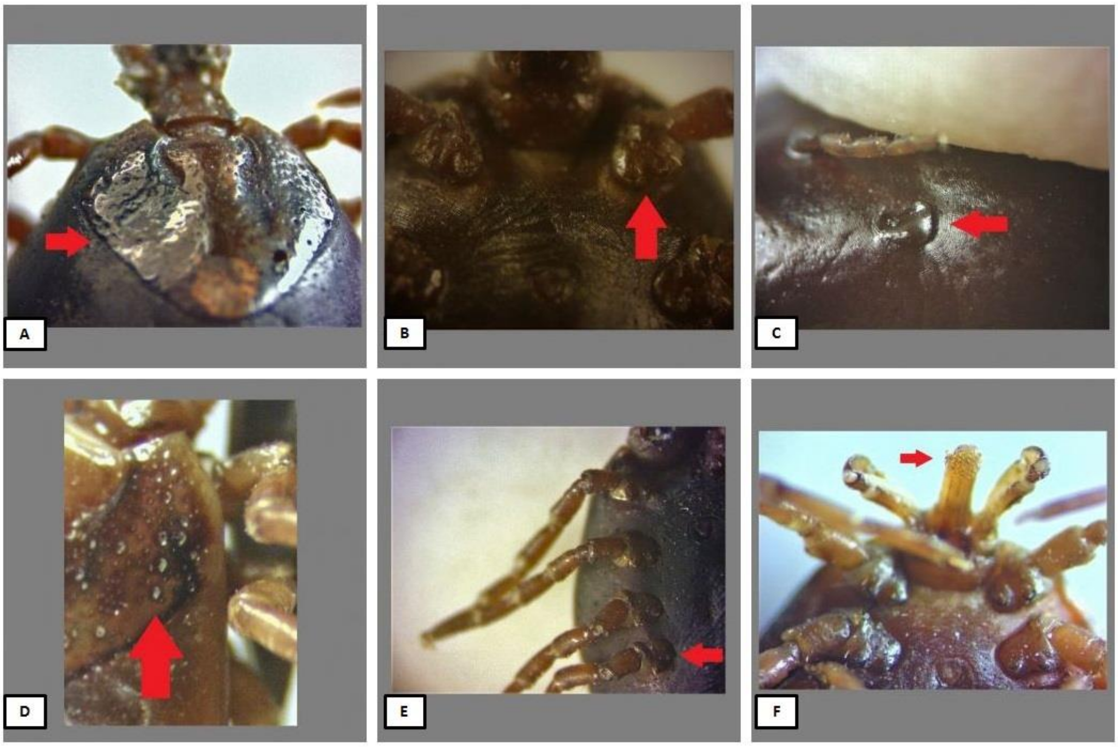

Supplementary Figure 1 – *Amblyomma rotundatum* female, morphological characteristics observed for species identification. The red arrows indicate the main characters evaluated during the identifications. (A) scutum cordiform, with a small, posterior pale spot, not extending to the midline of the scutum; (B) coxa I with two short rounded spurs; (C) spiracular plate; (D) scutum with numerous punctations in the anterolateral fields; (E) coxae I-IV with two short spurs; (F) hypostome dentition 3/3.
