# Supplementary material for: Ticks and Associated Pathogens From Rescued Wild Animals in Rainforest Fragments of Northeastern Brazil
Source: Front Vet Sci. 2020 Apr 8;7:177. doi: 10.3389/fvets.2020.00177 (PMC7179698; doi:10.3389/fvets.2020.00177)
Supplement: Supplementary file 2 [file Data_Sheet_2.PDF]

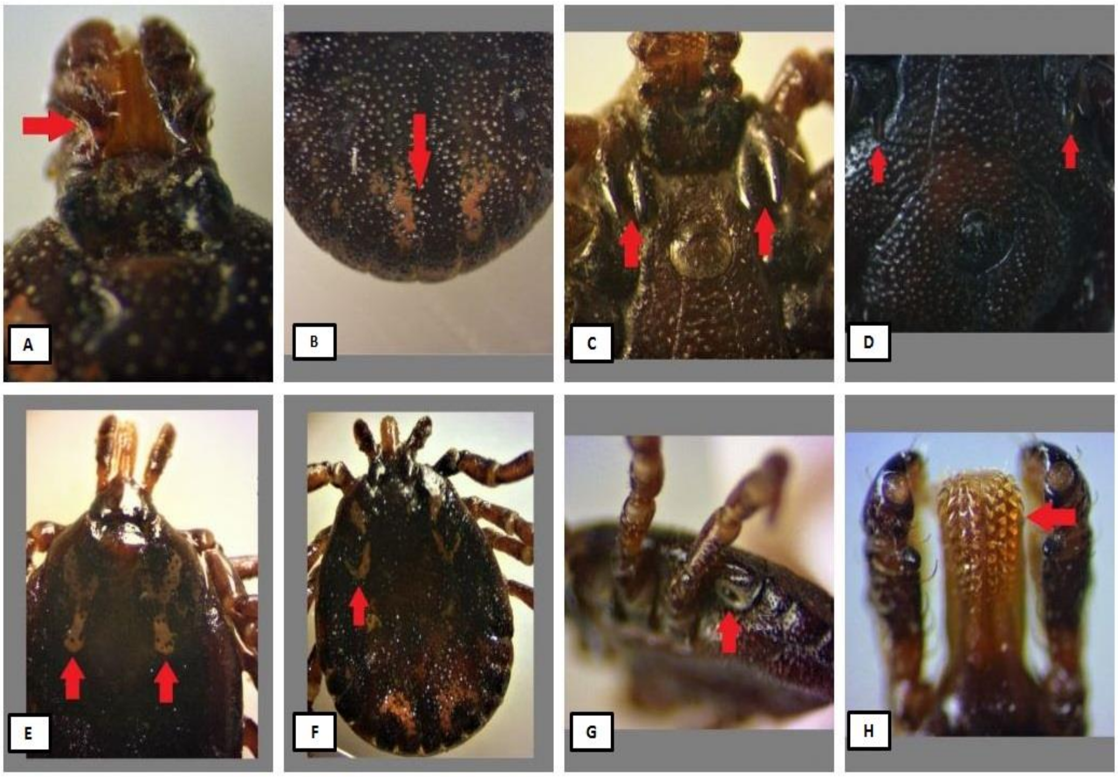

Supplementary Figure 2 – *Amblyomma nodosum* male and female, morphological characteristics observed for species identification. The red arrows indicate the main characters evaluated during the identifications. (A) male - palps short and nodose, article II with a dorsal oblique ridge; (B) male - marginal groove absent; (C) male - coxa I with two long, broad and subequal spurs, the internal slightly longer than the external; (D) male - coxa IV with a short spur; (E) female - scutum with symmetrically disposed, Y-shaped pale spots in the anterolateral fields; (F) male - scutum with two, symmetrically disposed, J-shaped pale spots in the anterolateral fields and other less pronounced, irregular spots; (G) male - spiracular plate comma-shaped; (H) female - hypostome dentition 3/3.
