# Supplementary material for: Ticks and Associated Pathogens From Rescued Wild Animals in Rainforest Fragments of Northeastern Brazil
Source: Front Vet Sci. 2020 Apr 8;7:177. doi: 10.3389/fvets.2020.00177 (PMC7179698; doi:10.3389/fvets.2020.00177)
Supplement: Supplementary file 3 [file Data_Sheet_3.PDF]

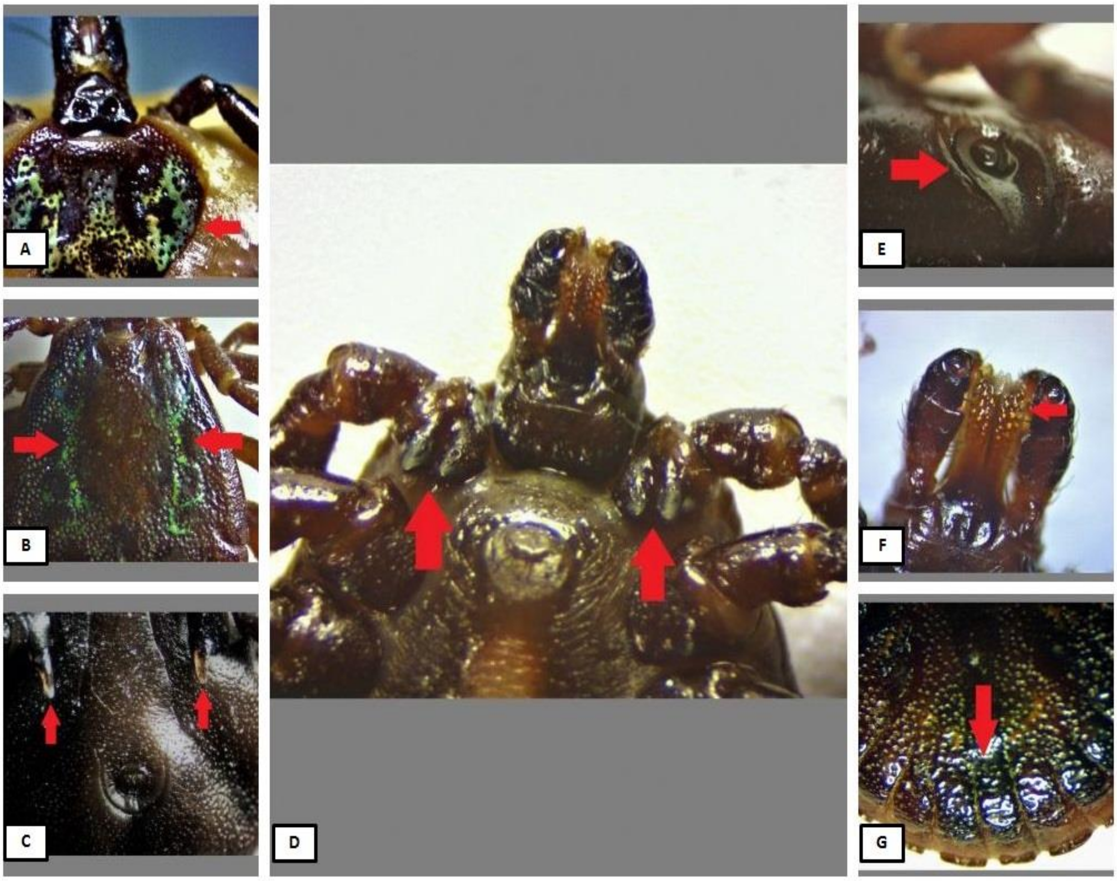


**Supplementary Figure 3 – *Amblyomma varium* male and female, morphological characteristics observed for species identification.** The red arrows indicate the main characters evaluated during the identifications. (A) female and (B) male - posterior and lateral fields of the scutum with irregular, coppery and greenish spots; (C) male - coxa IV with a long spur; (D) male - coxa I with two broad, subequal spurs, the external slightly longer than the internal; (E) male - spiracular plate comma-shaped (F) female - hypostome dentition 4/4; (G) male - marginal groove absent.
