# Supplementary material for: Ticks and Associated Pathogens From Rescued Wild Animals in Rainforest Fragments of Northeastern Brazil
Source: Front Vet Sci. 2020 Apr 8;7:177. doi: 10.3389/fvets.2020.00177 (PMC7179698; doi:10.3389/fvets.2020.00177)
Supplement: Supplementary file 4 [file Image_4.pdf]

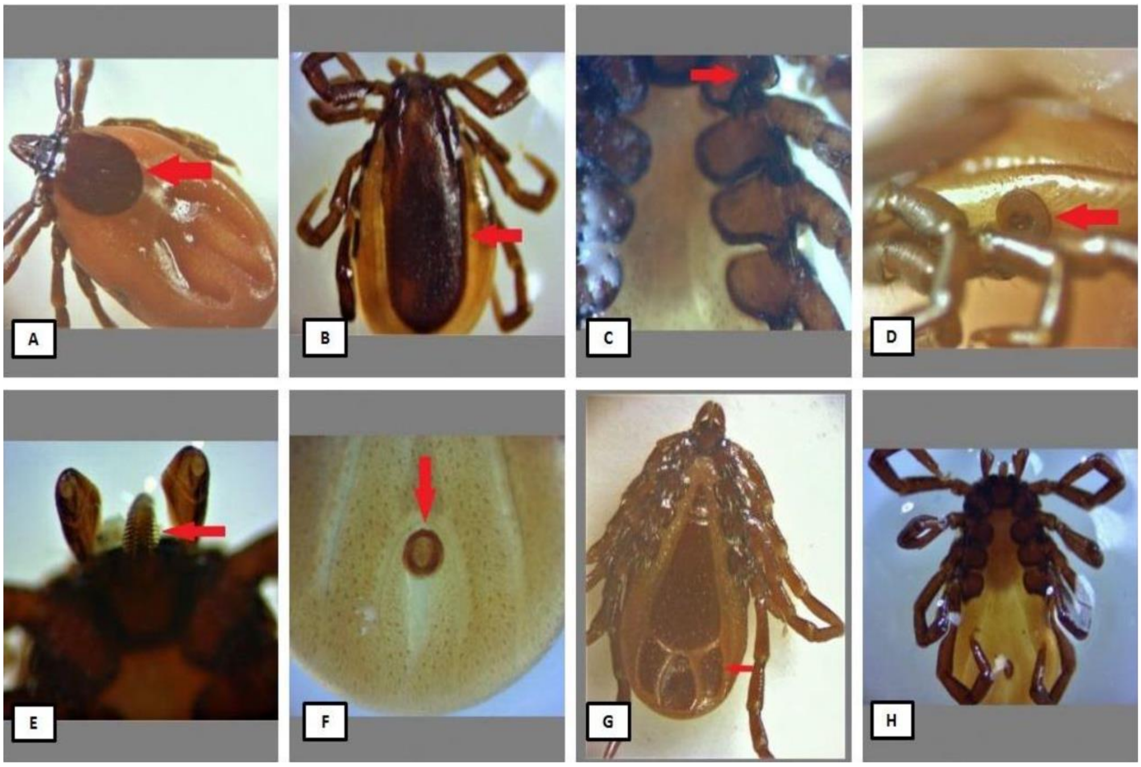

Supplementary Figure 4 – *Ixodes loricatus* male and female, morphological characteristics observed for species identification. The red arrows indicate the main characters evaluated during the identifications. (A) female – dorsal view, scutum oval and inornate; (B) male - dorsal view, scutum dark brown, inornate, with 2 anterolateral depressions extending to spiracular plates; (C) female - coxa I with two spurs, internal slightly smaller than external; (D) spiracular plate rounded; (E) female - hypostome 2/2 from base to apex, 3/3 at corona; (F) female - anal groove contouring the anus anteriorly; (G) male - ventral plates; ; (H) female - ventral view
