# Supplementary material for: Ticks and Associated Pathogens From Rescued Wild Animals in Rainforest Fragments of Northeastern Brazil
Source: Front Vet Sci. 2020 Apr 8;7:177. doi: 10.3389/fvets.2020.00177 (PMC7179698; doi:10.3389/fvets.2020.00177)
Supplement: Supplementary file 5 [file Image_5.pdf]

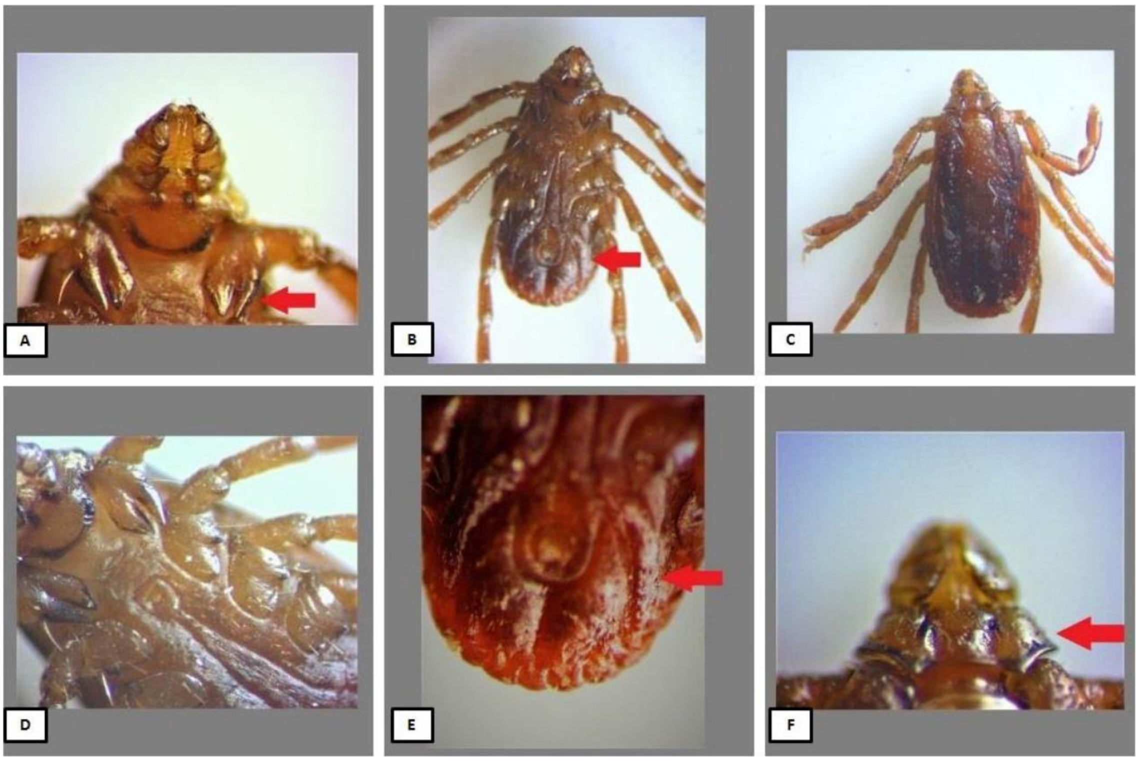

Supplementary Figure 5 – *Rhipicephalus sanguineus* female morphological characteristics observed for specie identification. The red arrows indicate the main characters evaluated during the identifications. (A) palps short and as long as the hypostome, dental formula 3/3; (B) ventral view; (C) dorsal view, scutum not densely pilose; (D) coxa I with two long and subequal spurs, coxae II-IV with short spurs; (E) anal groove distinct contouring the anus posteriorly; (F) basis capituli hexagonal dorsally.
